# Overnight emotional inertia in relation to depressive symptomatology and subjective sleep quality

**DOI:** 10.1093/sleepadvances/zpac048

**Published:** 2022-12-23

**Authors:** Liesse Frérart, Lauren Bilsen, Egon Dejonckheere, Peter Kuppens

**Affiliations:** Faculty of Psychology and Educational Sciences, KU Leuven, Leuven, Belgium; Faculty of Psychology and Educational Sciences, KU Leuven, Leuven, Belgium; Faculty of Psychology and Educational Sciences, KU Leuven, Leuven, Belgium; School of Social and Behavioral Sciences, Tilburg University, Tilburg, The Netherlands; Faculty of Psychology and Educational Sciences, KU Leuven, Leuven, Belgium

**Keywords:** emotional inertia, affect dynamics, negative affect, positive affect, sleep, depressive symptoms, sleep quality, experience sampling method

## Abstract

Emotions show a certain degree of continuity during the day, a quality referred to as emotional inertia, and that is typically elevated in depression. Little is known however about the extent to which our emotional experiences may or may not also persist overnight. Do our feelings continue from evening to morning or not? And how is this related to depressive symptoms and sleep quality? In an experience sampling studies in healthy subjects (*n*s = 123) we investigated (1) to what extent people’s mood, in terms of positive and negative affect, in the morning, after a night of sleep, can be predicted from their mood of the evening before, and whether this is moderated by (2) depressive symptom severity or (3) subjective sleep quality. Results showed that morning negative affect could be strongly predicted based on previous evening negative affect, whilst this carry-over effect was not observed for positive affect, indicating that negative affect shows a general tendency to persist overnight, while positive affect did not show such continuity. The overnight prediction of both negative and positive affect was not moderated by level of depressive symptoms, nor by subjective sleep quality.

**Statement of Significance**

What does a night’s sleep do to how we feel? Do our feelings continue, or do they not after a night’s sleep? This question is important to understand the impact of sleep on our emotional life, and how this may contribute to emotion disorder like in depression. Using smartphones, we measured people’s positive and negative feelings in the morning and evening, and examined how a person feels in the morning can be predicted by how they felt the evening before. We found carry-over effects from evening to morning for negative emotions, but not for positive emotions. These carry-over effects were unrelated to depressive symptom level or subjective sleep quality. Future research should be more considerate about this interplay between emotions and sleep.

How we feel emotionally throughout our daily lives seems to be mostly continuous in nature, as if one feeling state seamlessly blends into the experience of the next one. However, the only part of our lives where this may not be the case, is during our night’s sleep. When we wake up after a night’s sleep, do our emotions continue from the evening before, leading our emotional state from that time to be the best predictor of how we feel in the morning? Or is there no real relation between how we feel when we go to sleep and when we wake up the next morning? The answer to this question has crucial implications in light of understanding the interplay between emotion and sleep, as well as their role in mental health, and will be the central focus of this study.

## Overday and overnight emotional inertia

Emotional continuity is typically studied in terms of emotional inertia. Emotional inertia is defined as the degree to which a person’s current emotional state can be predicted by their previous emotional state (captured by the autocorrelation) [1]. When inertia or autocorrelation is low, a person’s previous emotional state is a bad predictor of their current emotional state. This implies that what happened in between these two moments influenced the (linear) trajectory of a person’s emotional state, signifying that their emotional state was altered by inside or outside events or stimuli [2]. However, when inertia or autocorrelation of emotions is high, a person’s previous emotional state is a very good predictor of a person’s current emotional state. This signifies that whatever happened in between these two moments has had relatively little impact on a person’s emotional state, as if decoupled from what contextually happened in between [1]. Inertia of emotions is generally found to be positive, yet important individual differences exist. Specifically, relatively higher levels of inertia have been related to emotional maladjustment or mental ill-health, like depressive symptoms [3].

The concept of emotional inertia has almost exclusively been studied during the daytime. Consequently, research on the topic of overnight emotions is very limited. Only recently, Minaeva et al. [4] investigated the difference between individuals who were either currently, never, or previously diagnosed with depression, in terms of their (1) overnight inertia for positive and negative affect, (2) the influence of sleep characteristics on overnight inertia and (3) the role of overnight inertia in predicting depression development. Yet, they did not focus on simple baseline levels of overnight inertia in their research questions. Moreover, their study was not designed for this research question, and therefore faced important methodological limitations (see below). As a result, we still know little about the basic continuity of emotions overnight. Therefore, in our primary research question we examine whether people’s mood in the morning, after a night’s sleep, can be predicted based on their mood of the evening before.

As far as we know, there is no direct empirical evidence addressing this question. It is also not known what the laymen’s opinion is on the continuity of overnight emotions. To address this, we performed a short survey in a convenience sample of 111 participants in which we asked whether positive and negative emotions tend to continue after a night’s sleep or rather return to a setpoint (see Supplementary Appendix A for details). The results showed that the layman is largely undecided on this question, with approximately half indicating continuity, half indicating none. So, it turns out that there is no lay consensus on how this question should be answered.

Second, we searched for indirect evidence to serve as a first lead, and we explored the bidirectional relation between emotion and sleep. To begin with, several studies have illustrated the influence of mood on sleep. Vandekerckhove et al. [5] showed that negative emotions in the evening, induced by daytime stressful events, increased sleep fragmentation and decreased sleep efficiency. Additionally, higher levels of repetitive thought in the evening, which often co-exist with negative mood, have been associated with increased sleep disturbance [6, 7]. In sum, this suggests that a bad evening mood is typically followed by worse sleep quality. Conversely, there is also research showing that sleep influences our mood. On the one hand, there is research indicating that bad sleep is commonly followed by a bad mood the next morning. In their review, Palmer and Alfano [8] typically found that decreased positive emotions and increased negative emotions were preceded by sleep loss. Also, slow wave activation in adults with bipolar disorder who were induced to feel sad, was related to impaired negative mood regulation the next morning [9]. Takano et al. [7] found that repetitive thought in the evening was associated with impaired sleep quality, which was in turn associated with reduced positive affect the next morning. On the other hand, there is also evidence indicating that sleep repairs mood. For instance, better sleep quality has been associated with decreased negative mood and increased positive mood the following day [10, 11]. As a possible mechanism for this mood repair function, Walker and van der Helm [12] hypothesized that sleep repairs mood the next day by decoupling the emotional component from an experience. It should be noted however that while this evidence and theorizing is informative, it pertains to the total day mood, instead of the mere morning mood. Therefore, this literature is less relevant for investigating morning mood.

This brief overview of indirect evidence suggests that mood has an effect on sleep and sleep has an effect on mood. Based on this, it could be expected that mood will show a certain continuity overnight, where a person who feels relatively good in the evening will also feel relatively good in the morning, and vice versa.

## The role of depressive symptomatology and sleep quality

As our second and third research questions, as a replication of the results reported by Minaeva et al. [4], we will examine the moderating role of depressive symptoms and sleep quality in overnight emotions, respectively. Regarding depressive symptoms, as mentioned, this has been found to be related to higher levels of (overday) emotional inertia. In general, less flexible emotional responding has been associated with psychological maladjustment [13]. Emotions are highly dynamic, allowing us to quickly and efficiently adapt to internal and external events [14]. However, when our emotions become less dynamic (i.e. when emotional inertia is high), they can no longer adapt to current challenges and opportunities. Such decreased adaptation, akin to what Rottenberg [15] labeled as *increased emotion context insensitivity*, is a documented characteristic of depression, meaning that people with depressive symptoms show reduced emotional reactivity to their environment [16]. Indeed, higher levels of depressive symptoms have been associated with higher levels of inertia, regarding both positive and negative emotions. In other words, for people with higher levels of depressive symptomatology, both positive and negative emotional states are slower to change compared to people with lower levels of depressive symptoms (e.g. studies by Kuppens et al. [3] and Koval et al. [17]; for a meta-analysis, see Houben et al. [18], but also see Dejonckheere et al. [19] for a cautionary note). The question in the current context is therefore whether high overnight inertia, is also higher in people with more depressive symptoms. In the study by Minaeva et al. [4], depression status moderated overnight inertia with this being higher in the currently depressed group compared to the non-depressed group, when controlling for sleep quality and duration. However, no such association was found for positive affect. This suggest that level of depression only directly impacts the continuity of overnight negative affect in clinically depressed individuals. It could be that depression only starts interfering with sleep and emotional continuity after exceeding a certain threshold of depression severity level. Additionally, Lewinsohn et al. [20] found that 28% of adolescents with a history of MDD reported depressed mood without anhedonia, and only 5% experienced anhedonia without depressed mood. In light of these findings, they posed that depressed mood might play a more pivotal role in MDD than anhedonia. Knowing that depressed mood is part of the broad construct of negative affect [21], this may be why depression impacts negative affect more than positive affect. As a result, following the findings by Minaeva et al. [4], but contrary to the literature on overday emotions, we expect that level of depressive symptoms will not moderate overnight inertia in a relatively healthy sample.

Regarding sleep, as mentioned above, Takano et al. [7] showed that impaired sleep quality was associated with reduced positive affect the next morning, which in turn was associated with increased repetitive thoughts (i.e. reduced positive affect) the next evening. Åkerstedt et al. [22] found that bedtime stress and worries were two of the main predictors of subsequent sleep quality. These studies suggest that evening mood influences sleep quality, which in turn influences morning mood. In the current context of overnight inertia, we examine whether after a night with worse sleep quality, emotional inertia of negative affect is higher and emotional inertia of positive affect is lower. Minaeva et al. [4] found no significant association between overnight affective inertia and sleep quality, both for clinically depressed participants and healthy controls. Since Minaeva et al. [4] directly investigated the influence of sleep quality on overnight inertia, contrary to the rest of the literature which only provides indirect evidence, we expect to find no moderation of sleep quality on overnight emotional inertia.

## The Current Study

In the first research question we will verify to what extent people’s mood in the morning, after a night of sleep, in terms of positive and negative affect, can be predicted based on their mood of the evening before across different days of measurement (e.g. within-person prediction). To interpret the size of the prediction, we will compare this prediction to the opposite scenario, where evening mood is predicted by that same day’s morning mood across different days of measurement. This overday prediction spans a similar time difference as overnight emotions, with the important difference that people live an awake life in between with all types of possible emotional stimulation one encounters. Our second research question is whether the morning mood of people with higher levels of depressive symptoms can be predicted more strongly based on their mood in the evening (showing higher levels of overnight inertia), compared to people with lower levels of depressive symptoms. Our third research question is whether after a night with worse self-reported sleep quality, negative affect will be predicted more strongly and positive affect less strongly, whereas after a night with better self-reported sleep quality, positive affect will be predicted more strongly and negative affect less strongly.

To answer these questions, participants’ level of depressive symptoms were assessed at baseline by means of the Center for Epidemiologic Studies Depression Scale (CES-D) [23]. Next, participants were asked, using the Experience Sampling Method (ESM) [24], to indicate their emotions two times a day, once in the morning and once in the evening, and this for a total duration of 14 consecutive days. In the context of ESM, such a single measurement occasion is referred to as a *beep*. Additionally, every morning, participants indicated their subjective sleep quality of the past night. Based on this data, we examined the within-person prediction of morning mood by evening mood (and vice-versa), and whether this is moderated by person-level depression severity and day-level subjective sleep quality.

Although Minaeva et al. [4] provided first valuable insights in overnight emotional inertia and its relationship with depression, the current study goes beyond that of Minaeva et al. [4] in several important ways. First, Minaeva et al. [4] focused on overnight inertia in relation to depression in each of their research questions, sidestepping our first research question. Second, they studied an exclusively female sample. Third, they only sampled observations from 5 consecutive days, which results in a very low maximally possible number of overnight observations per participant, leading to possibly unreliable estimates. Fourth, Minaeva et al. [4] included multiple beeps between the first and last beep of the day, perhaps creating an increased continuity in participants’ experience due to the fact that they were reporting on their affect several times in a row during the day, which is absent during the night, hindering a clear-cut comparison. We replicate and extend their study by, first, taking a step back and investigating base-level emotional inertia in itself. Second, we will make use of more gender-balanced samples. Third, we sampled 14 consecutive days. Fourth, participants did not complete additional measurement occasions between the morning and evening assessments, which provides a proper comparison of overday and overnight inertia.

Concretely, we investigated separately for positive and negative affect to what extent: (1) a person’s evening affect can be predicted based on his/her morning affect (across all days; meaning within-person prediction), (2) a person’s morning affect can be predicted based on his/her previous evening affect (across all days; meaning within-person prediction), (3) there is a difference between the prediction after a night compared to a day (cfr. research question 1), and (4) this is a function of depressive symptoms (cfr. research question 2) or (5) subjective sleep quality (cfr. research question 3).

## Methods

### Participants

Participants were recruited by means of a public message on Facebook, asking for participants in a study on sleep and emotions (without providing further information on the purpose or research questions behind the study; e.g. there is no mention of continuity or overday or overnight emotions). This resulted in a sample of 127 Dutch-speaking participants, located all over Flanders, Belgium. Four participants were excluded because their compliance was less than 50%, therefore the final sample consisted of 123 participants. All participants had a chance to win one of five gift vouchers of Bol.com, worth 20 euros each. This study was approved by “Privacy and Ethical Review” (PRET; G-2020-2010). Descriptive statistics are presented in Table 1.

**Table 1.** Descriptive statistics of sample and all used measures

Measure	Score	Morning score	Evening score
Sex, *n* (%)			
Men	33 (26.83)		
Women	88 (71.54)		
Non-binary	2 (1.63)		
Age, mean (*SD*)	30.70 (14.35)		
Happy, mean (*SD*)	60.84 (15.32)	60.32 (14.29)	61.34 (15.63)
Relaxed, mean (*SD*)	65.60 (17.50)	63.32 (16.71)	67.88 (16.94)
Energetic, mean (*SD*)	46.25 (19.43)	49.51 (16.71)	42.94 (18.68)
Peaceful, mean (*SD*)	65.76 (15.51)	65.00 (14.66)	66.47 (15.63)
Enthusiastic, mean (*SD*)	55.04 (17.05)	55.68 (15.42)	54.26 (17.63)
Satisfied, mean (*SD*)	64.71 (15.53)	64.02 (14.27)	65.37 (15.97)
Angry, mean (*SD*)	15.44 (13.26)	15.22 (12.13)	15.62 (13.44)
Down, mean (*SD*)	23.44 (16.12)	23.37 (14.83)	23.52 (16.29)
Unsatisfied, mean (*SD*)	26.86 (16.67)	27.30 (15.56)	26.47 (16.94)
Stressed, mean (*SD*)	32.12 (18.86)	34.24 (18.56)	30.02 (18.21)
Tired, mean (*SD*)	49.73 (22.35)	46.48 (20.01)	53.00 (20.07)
Sad, mean (*SD*)	23.00 (15.39)	22.54 (14.29)	23.41 (15.36)
Positive affect, mean (*SD*)	59.70 (12.82)	59.64 (11.94)	59.71 (12.83)
Negative affect, mean (*SD*)	28.43 (12.02)	28.19 (11.30)	28.67 (11.86)
CES-D, mean (*SD*)	14.25 (10.71)		
Subjective sleep quality, mean (*SD*)		3.38 (0.62)	

CES-D, Center for Epidemiological Studies Depression scale.

### Procedure

Because of the COVID-19 pandemic, the entire procedure was carried out online. To begin with, participants received a private message or an e-mail containing the informed consent and the instructions to install and configure the m-Path app [25]. They were asked to read and sign the informed consent and to return a scan or picture of the signed document. After installing the app, participants were presented with a demographics questionnaire, asking for their age, gender, and e-mail address, together with the CES-D and another questionnaire not used in this study. Participants were assured that the sole use of the e-mail address was that of sending the gift voucher in case they won. From then on, participants received two beeps a day, one in the morning and one in the evening, with 12 h in between (*M*_interval_ = 11.82 h; as such, the interval in between two measurements was kept constant and identical for overday and overnight prediction, even though the exact timing could be chosen by the participant to fit their daily routine), for a duration of 14 days. Participants were able to choose the daily start and end times of the ESM sampling schedule that corresponded best with their sleep schedule, in order to reduce the time difference between the scheduled beeps and participants’ wake up and sleep time. Compliance was high (*M* = .90; *SD* = .16). After completion, participants were debriefed about the main goals of the research and thanked for their participation.

### Materials

#### CES-D.

The CES-D [23] was administered to assess depressive symptomatology (range = 0–52). The questionnaire consisted of 20 items, asking how many times participants felt or behaved a certain way over the past week, for example: “I had crying spells”. The four answer possibilities included: “Rarely or none of the time (Less than 1 day)”, “Some or a little of the time (1–2 days)”, “Occasionally or a moderate amount of time (3–4 days)” and “Most or all of the time (5–7 days)”. Scores on the CES-D could range between 0 and 60, where a higher score is indicative of more depressive symptoms. The CES-D showed excellent reliability, with an internal consistency of *α* = .93.

#### ESM questionnaire.

The ESM questionnaire asked participants to indicate on a slider ranging from 1 (“Not at all”) to 100 (“Very much”) how much a certain emotional label applied to their state at the moment they received the beep. Based on the 12-Point Circumplex Structure of Core Affect, construed by Yik et al. [26], the 12 emotional labels included: *happy*, *relaxed, energetic, peaceful, enthusiastic, satisfied, angry, down, unsatisfied, stressed, tired, and sad.* The mean score of the first six emotions resulted in a measure of positive affect, whereas the mean score of the latter six emotions resulted in a measure of negative affect. Within-person reliability of positive affect was .77, whereas the between-person reliability was .95. For negative affect, the within-person reliability was .56, whereas the between-person reliability was .96. Additionally, the first beep of the day asked to indicate their sleep duration on a slider ranging from 1 (‘Enough’) to 4 (“Very little/not at all”) and sleep quality on a slider ranging from 1 (‘Good’) to 4 (“Very bad/not at all”), both derived from the Athens Insomnia Scale (AIS) [27]. The item about sleep quality results indeed in a subjective assessment of sleep quality and may be more prone to bias. However, such one-item measure of sleep quality showed having sufficient criterion validity [28], while being more practical for using in ESM studies as opposed to, for example, polysomnography. Moreover, sleep quality has been associated with measures of health and well-being, such as feelings of tension, depression, confusion, and fatigue [29]. The first beep of the day also asked whether participants dreamt and if so, if the dream had a positive or negative emotional load. The items about sleep duration and dreams were not further included in our analysis (however, results of multilevel analyses regarding moderation by sleep duration can be found in Supplementary Appendix B).

## Analytic Strategy

Given the nested nature of the data, namely beeps nested in persons, we performed multilevel analyses using the lme4 package for R [30]. To be able to fit the models we specified to test our predictions (see below), the data were preprocessed in the following manner. Firstly, we created two lagged variables from positive affect and negative affect. Secondly, two dummy variables were created. *Dummy evening* (E) specified whether a beep occurred in the evening (1), or not (0), whereas *dummy morning* (M) specified whether a beep occurred in the morning, after a night of sleep (1), or not (0). By introducing a dummy variable for each category (day and night), the coefficients are each interpreted as testing whether they are different from 0 (i.e. estimation), as opposed to traditional *n*-1 dummy coding where they are interpreted as being relative to a baseline (i.e. hypothesis testing). In the multilevel models, the level-1 predictors were person-mean centered and level-2 CES-D scores were standardized. Significance was tested against an alpha of 0.05. The descriptive statistics of all used measures are presented in Table 1.

For both positive and negative affect, we constructed a series of five linear multilevel (mixed-effect) models to test our predictions (see Supplementary Appendix C for a detailed description and equations of the models). In these models, (evening and morning) affect is predicted by previous (morning and evening) affect within-person across all measured days. The (dummy) intercept and slope parameters in the models are allowed to vary across individuals by means of random effects. To answer our primary research question, in the first model, it was tested to what extent affect is self-predictive during the day by assessing the interaction effect between dummy evening and the affect of the previous observation (Model 1). In terms of interpretation, a significant interaction effect would provide evidence for continuity or self-predictability of affect during the day. A non-significant interaction effect, in turn, would mean that there is no significant relationship between morning and evening affect. In a second model, it was tested whether someone’s affect after a night’s sleep can be predicted by the last point of the previous day by assessing the effect of the interaction between dummy morning and the affect of the previous observation (Model 2). Likewise, in terms of interpretation, the presence of a significant interaction would be an indication of self-predictability or continuity of affect overnight, as affect from the previous evening would be a significant predictor of affect in the morning. In contrast, if this overnight prediction would not be significant, it indicates that there is no relationship between evening and morning affect. In a third model, Model 1 and Model 2 were combined to be able to compare the prediction of affect overnight to the prediction of affect during the day and to assess possible diurnal effects (Model 3). Regarding our second research question, in a fourth model, it was tested whether level of depressive symptoms moderated the extent to which affect is self-predictive during the day by examining the effect of the interaction between dummy evening, the affect of the previous observation and level-2 CES-D scores (Model 4). In a fifth model, it was tested whether level of depressive symptoms moderated the extent to which affect is self-predictive overnight by assessing the interaction effect of dummy morning, the affect of the previous observation and level-2 CES-D scores (Model 5). To answer our third research question, in a sixth model, we tested whether subjective sleep quality moderates the extent affect is self-predictive during the day by examining the interaction effect of dummy evening, the affect of the previous observation and the self-reported sleep quality scores (Model 6). Lastly, in a seventh model, it was tested whether subjective sleep quality relates to what extent affect is self-predictive overnight by assessing the effect of the interaction between dummy morning, the affect of the previous observation and the self-reported sleep quality scores (Model 7).

## Results

An overview of the results of the prediction of negative and positive affect using Models 1–7 are provided in Table 2.

**Table 2.** Multi-level models estimating mean level and autoregressive effect during day and night, as a function of affective state

Fixed effects	Negative affect		Positive affect	
	*β *(*SE*)	*P*	*β *(*SE*)	*P*
Model 1: Overday affect				
Mean affect	28.56 (1.23)		60.09 (1.19)	
Autoregressive effect	0.23 (0.06)	<.001	0.13 (0.09)	.14
Model 2: Overnight affect				
Mean affect	27.95 (1.24)		59.88 (1.25)	
Autoregressive effect	0.25 (0.06)	<.001	0.16 (0.09)	.08
Model 3: Comparison overnight to overday affect				
Mean overday affect	28.68 (1.22)		59.88 (1.17)	
Autoregressive effect overday	0.22 (0.04)	<.001	0.19 (0.03)	<.001
Mean overnight affect	27.91 (1.21)		59.81 (1.16)	
Autoregressive effect overnight	0.19 (0.04)	<.001	0.16 (0.03)	<.001
Model 4: Moderation CES-D on overday affect				
Mean affect	28.35 (0.93)		60.26 (1.16)	
Autoregressive effect	0.21 (0.06)	<.001	0.13 (0.09)	.17
Mean CES-D	8.85 (0.92)		−7.14 (1.15)	
CES-D × overday affect	−0.02 (0.05)	.65	−0.05 (0.08)	.51
Model 5: Moderation CES-D on overnight affect				
Mean affect	27.78 (0.92)		60.09 (1.24)	
Autoregressive effect	0.24 (0.06)	<.001	0.16 (0.09)	.09
Mean CES-D	9.00 (0.91)		−7.79 (1.22)	
CES-D × overnight affect	−0.01 (0.05)	.79	0.02 (0.08)	.81
Model 6: Moderation sleep quality on overday affect				
Mean affect	28.67 (1.24)		59.92 (1.21)	
Autoregressive effect	0.22 (0.06)	<.001	0.11 (0.10)	.25
Mean sleep quality	0.20 (1.36)		−0.12 (2.43)	
Sleep quality × overday affect	−0.04 (0.12)	.76	0.04 (0.19)	.82
Model 7: Moderation sleep quality on overnight affect				
Mean affect	27.83 (1.23)		59.90 (1.30)	
Autoregressive effect	0.23 (0.06)	<.001	0.14 (0.10)	.16
Mean sleep quality	1.20 (1.46)		−0.11 (2.60)	
Sleep quality × overnight affect	0.04 (0.12)	.75	0.07 (0.19)	.70

Level-1 predictors were person-mean centered. CES-D scores were standardized; CES-D, Center for Epidemiological Studies Depression scale.

### Prediction of negative affect

First, we found that negative affect in the morning significantly predicts negative affect in the evening (Model 1), and vice-versa, that negative affect in the evening significantly predicts negative affect the next morning (Model 2). These results imply that negative affect is self-predictive, both after a day and a night’s sleep, showing evidence for continuity of negative affect. The difference between the autoregressive effect after a day and after a night’s sleep was not significant (Model 3; *z* = 0.63, *p* = .53), suggesting that there is not significantly more or less continuity of negative affect during the night than during the day. In addition, the difference between dummy evening and dummy morning was not significant (*z* = 1.12, *p* = .26), indicating that no significant diurnal effects were found for negative affect.

Second, regarding the moderation by level of depressive symptoms, no significant interaction was found between the autoregressive effect of negative affect and CES-D scores, neither after a day (Model 4), nor after a night’s sleep (Model 5). This indicates that the prediction of negative affect is not stronger for individuals with higher levels of depressive symptoms.

Third, regarding the moderation by subjective sleep quality, the interaction between the autoregressive effect of negative affect and sleep quality was not significant, both after a day (Model 6) and after a night’s sleep (Model 7). These results taken together suggest that there is no association between negative affect and sleep quality.

### Prediction of positive affect

Firstly, no significant effect of positive affect was found, neither after a day (Model 1), nor after a night’s sleep (Model 2). Combined, these results suggest that positive affect is not continuous during both day and night. However, when predicting positive affect with the combined model (Model 3), a significant effect was found during the day, as well as after a night’s sleep. Moreover, continuity of positive affect during the night and during the day did not significantly differ (*z* = 0.70, *p* = .49). The difference between dummy evening and dummy morning was not significant (*z* = 0.89, *p* = .93), indicating no significant diurnal effects.

Secondly, regarding the moderation by level of depressive symptoms, the interaction effect between CES-D scores and the autoregressive effect of positive affect during the day (Model 4) and after a night’s sleep (Model 5) were both not significant. This suggests that positive affect is not more continuous in individuals with higher levels of depressive symptoms.

Thirdly, regarding the moderation by subjective sleep quality, no significant interaction was found between subjective sleep quality and positive affect, both after a day (Model 6) and a night’s sleep (Model 7). These results combined, suggest that there is no significant association between positive affect and subjective sleep quality after a day, as well as after a night’s sleep.

## Discussion

This study aimed to investigate, first, whether across multiple days people’s mood in the morning, after a night of sleep, can be predicted based on their mood of the evening before. Second, we examined whether people with higher levels of depressive symptoms’ mood in the morning, can be predicted more strongly based on their mood in the evening, compared to people with lower levels of depressive symptoms. Third, we examined whether after a night with worse self-reported sleep quality, negative affect can be predicted more strongly and positive affect less strongly, whereas after a night with better self-reported sleep quality, positive affect can be predicted more strongly and negative affect less strongly.

Regarding our primary research question, we found evidence to conclude that people’s negative affect in the morning, after a night’s sleep, can be predicted based on their negative affect from the evening before, implying that negative affect shows continuity overnight in a sample of healthy individuals. The same cannot be concluded when it comes to positive affect, however. Namely, after a night’s sleep, positive affect does not strongly predict itself based on the previous evening, signifying that positive affect is not continuous overnight. These conclusions remain the same when considering daytime inertia. This result for overnight negative emotions links up with literature indicating that emotions tend to color our experiences and perceptions of the outside world in a mood-congruent way. Although this has been found for both positive and negative emotions (e.g. [31–33]), this might be more pronounced for the latter [34, 35]. At the brain-level, negative mood facilitates processing of negative stimuli or information by inducing changes in the organization of neural networks involved in guiding our interaction with the external world. For instance, negative affect inertia has been linked to inflexible activity and elevated connectivity of the DMN, associated with self-referential emotional processing [36]. This altered DMN organization might cause more resources to be attributed to internal processes at the cost of external emotional cues [36], biassing our perception of the environment towards negative mood-congruent stimuli. The absence of observed continuity of positive affect can perhaps be explained by the notion that positive affect exhibits relatively stronger circadian variability than negative affect [37], as will be further explained below.

Regarding the second research question, no moderation effect of level of depressive symptoms on the continuity of both positive and negative affect was found, neither during the day, nor after a night’s sleep. Specifically, people with higher levels of depressive symptoms’ mood in the morning, cannot be predicted more strongly based on their mood in the evening, compared to people with lower levels of depressive symptoms. This could also be inferred from the study by Gordijn et al. [38], who reported no significant association between depression severity, mood variability, and diurnal variability. Perhaps, the expected moderation effect of depressive symptoms was not found, because our sample did not include patients diagnosed with major depressive disorder, possibly resulting in less variance in the collected CES-D scores over participants. This notion can be substantiated by the findings of Minaeva et al. [4], where moderation of overnight negative affect by depression status was only seen for clinically depressed individuals and not in healthy individuals.

Regarding the third research question, the results did not indicate a moderation effect of sleep quality on the continuity of neither negative nor positive affect, both during the day and after a night. This means that after a night with worse self-reported sleep quality, negative affect will not be predicted more strongly and positive affect will not be predicted less strongly, whereas after a night with better self-reported sleep quality, positive affect will not be predicted more strongly and negative affect will not be predicted less strongly. This is again in line with what we expected based on the findings of Minaeva et al. [4]. Indeed, as Minaeva et al. [4] posed, subjective measures of sleep quality may yield different implications as opposed to more objective measures. Unlike in our results, such moderation effects have previously been reported in people with Insomnia Disorder. In one study, the physical component of induced shame increased after a night’s sleep, whilst it decreased in normal sleepers. However, no such effect was found for the emotional and social component of shame [39]. In another study, frequently interrupted REM sleep was found to inhibit overnight resolution of emotional distress by impeding amygdala adaptation [40]. Above differences in results might be due to the fact that sleep quality was defined as whether or not participants suffered from insomnia disorder or the frequency of REM sleep perturbations, while we looked at subjective sleep quality in a healthy sample.

Even though the current study accounts for several of the limitations of previous research, it has certain limitations as well. Firstly, while a 12 h time period in between observations was chosen to enhance the comparability of overday and overnight emotion prediction, in fact people are usually awake for more than 12 h a day. It is therefore possible that an event with a considerable emotional impact happened before the first observation in the morning, or after the last observation in the evening, influencing subsequent emotions. Future studies should take more care of making sure that participants report on their affective state right before going to sleep, and as soon as possible after they wake up. Secondly, we examined a relatively healthy, young, western sample of participants. While Minaeva et al. [4] accounted for this limitation, by including clinically depressed groups, future research is needed to examine whether overnight emotions take on a different shape in physically or mentally less healthy, older, or non-western populations.

It is necessary to point out that in the current study we did not consider (individual differences in) circadian rhythms, which have previously been found to interact with mood regulation [41]. For instance, previous research showed that positive affect amplitude decreases along the continuum from morning to evening chronotypes, whereas no such associations were found regarding negative affect [42]. Consequently, an interesting future hypothesis might be that positive affect is more continuous overnight in evening types, compared to morning types, whilst negative affect might not show such moderation by chronotype. Additionally, positive affect has been found to display relatively stronger circadian variability, compared to negative affect [37]. It could be worthwhile to examine to what extent circadian rhythms interact with overnight and overday inertia in order to provide more insight into the found pattern of results. Also, further investigating the potential role of the intensity of emotions or the emotional load of dreams [43, 44] in the phenomena of overnight inertia will add great value to the research field.

In conclusion, in this study we asked the question whether our emotional experience in the evening continues after a night’s sleep. The answer to that question in healthy subjects is that negative affect seems to persist overnight to at least some extent, whereas positive affect more likely resets, and these findings are independent of sleep quality or depressive symptom level.

## Supplementary Material

zpac048_suppl_Supplementary_Appendix

## Data Availability

The data underlying this article will be shared on reasonable request to the corresponding author.
