# Supplementary material for: Overnight emotional inertia in relation to depressive symptomatology and subjective sleep quality
Source: Sleep Adv. 2022 Dec 23;4(1):zpac048. doi: 10.1093/sleepadvances/zpac048 (PMC10108641; doi:10.1093/sleepadvances/zpac048)
Supplement: zpac048_suppl_Supplementary_Appendix [file zpac048_suppl_supplementary_appendix.docx]

# Appendix A

# Overnight Inertia According to the Layman

Since there is no clear answer to our research question based on the literature, we were curious what people’s implicit theories on the topic are. Therefore, we asked the layman’s opinion on the continuity of overnight emotions, using a short questionnaire.

## Methods

### Participants

All participants were part of a convenience sample from the entourage of researchers, and were recruited via private groups or private messages on Facebook. The sample that responded to our invitations consisted of 111 Dutch-speaking participants (31 men, 80 women; *M_age_* = 28.64, *SD_age_* = 13.12).

### Materials

The questionnaire surveyed participants’ experiences regarding their sleep and emotions, by means of a short online form. Participants were asked to think about the last time they experienced the questioned emotion. The form consisted of two Dutch multiple-choice questions, constructed by the researchers. The first question asked about participants’ experience regarding positive emotions in the morning after a night’s sleep (e.g., ‘*When you experienced a positive emotion in the evening, was your emotion the next morning:*’). The second question asked about their experience regarding negative emotions in the morning after a night’s sleep (e.g., ‘*When you experienced a negative emotion in the evening, was your emotion the next morning:*’). For both questions, participants had to choose between two possible answers. If they indicated the first option (e.g., for the first question: ‘*Still positive, about the same as the night before'*), they thought their emotion stayed the same as before they went to sleep. If they indicated the second option (e.g., for the first question: ‘*Reset, neutral again’*), they thought their emotions were reset to a setpoint.

### Procedure

Participants were informed that their participation remained completely anonymous. After filling out their sex and age, participants were presented with the two above mentioned questions. Last, participants had the possibility to leave a remark. The entire procedure took approximately five minutes.

## Results

For positive emotions, 45 (40.54%) participants thought their emotions stayed the same and 66 (59.46%) participants thought their emotions were reset to a setpoint. For negative emotions, 58 (52.25%) participants thought their emotions stayed the same and 53 (47.75%) participants thought their emotions were reset to a setpoint.

To test whether there is agreement between participants, an exact binomial significance test was conducted. The test revealed no significant difference between the two possible answers for both positive, *p* = .06, 95% CI [.50, .69], and negative emotions *p* = .57, 99% CI [.35, .59]. So, based on this test, there is no agreement among the layman on whether their emotions stayed the same over the night or whether their emotions were reset to a setpoint.

## Discussion

We conclude that people do not agree on whether both their positive and negative emotions in the morning are reset to a setpoint or stay more or less the same level as before a night’s sleep. In other words, there is no agreement among the layman on what the intuitive answer to our research question might be: roughly half of our participants thought that their emotions in the evening stayed the same in the morning, after a night’s sleep, whereas the other half of our participants believed that their emotions were set to a reset point after a night’s sleep.

# Appendix B

# Results of Multilevel Analyses Moderation by Sleep Duration

## Results

Regarding the moderation by sleep duration, the interaction between the autoregressive effect of both positive and negative affect and sleep duration was not significant, neither after a day, nor after a night’s sleep. These results taken together suggest that there is no association between both positive and negative affect and sleep duration.

**Table C1**

*Multi-Level Models Estimating Mean Level and Autoregressive Effect During Day and Night, as a Function of Affective State.*

| Fixed Effects | Negative Affect | | Positive Affect | |
| --- | --- | --- | --- | --- |
|  | β (*SE*) | p | β (*SE*) | p |
| *Moderation Sleep Duration on Overday Affect* | | | | |
| Mean affect | 28.69 (1.24) |  | 59.91 (1.21) |  |
| Autoregressive effect | 0.22 (0.06) | <0.001 | 0.11 (0.10) | 0.25 |
| Mean Sleep Duration | 1.09 (1.61) |  | -1.05 (2.90) |  |
| Sleep Duration x overday affect | 0.01 (0.14) | 0.94 | 0.12 (0.23) | 0.62 |
| *Moderation Sleep Duration on Overnight Affect* | | | | |
| Mean affect | 27.89 (1.23) |  | 59.91 (1.30) |  |
| Autoregressive effect | 0.24 (0.06) | <0.001 | 0.14 (0.09) | 0.14 |
| Mean Sleep Duration | 0.10 (1.69) |  | 1.06 (3.00) |  |
| Sleep Duration x overnight affect | 0.03 (0.14) | 0.81 | 0.10 (0.23) | 0.65 |

*Note.*Level-1 predictors were person-mean centered.

# Appendix C

# Detailed Description of the Models

## Model 1

In a first model, we tested to what extent affect is self-predictive during the day. This model was estimated using the following equation:

$Y_{ij}=\beta_{1i}*E_{ij}+\beta_{2i}*E_{ij}*X_{ij-1}+\varepsilon_{ij}$

*Eq 1*

$Y_{ij}$: predicted affect for person i at time j

$X_{ij-1}$: affect of person i at time j-1

$E_{ij}$: dummy evening; all beeps that occurred in the evening, not after a night’s sleep

$\beta_{1i}$: slope of dummy evening, or intercept for all beeps which occurred in the evening, not after a night’s sleep

$\beta_{2i}$ : the slope of the lagged affect of a person at a certain beep for beeps that occurred in the evening

$\varepsilon_{ij}$: error component

As you can see, Model 1 tests whether someone’s affect at one point of the day can predict their affect at the next point of the day.

## Model 2

In a second model, we tested to what extent affect is self-predictive overnight. This model was estimated using the following equation:

$$Y_{ij}=\beta_{1i}*M_{ij}+\beta_{2i}*M_{ij}*X_{ij-1}+\varepsilon_{ij}$$

*Eq 2*

$Y_{ij}$: predicted affect for person i at time j

$X_{ij-1}$: affect of person i at time j-1

$M_{ij}$: dummy morning; all beeps which occurred in the morning, after a night’s sleep

$\beta_{1i}$: slope of dummy morning, or intercept for all beeps which occurred in the morning, after a night’s sleep

$\beta_{2i}$ : the slope of the lagged affect of a person at a certain beep for beeps that occurred in the morning, after a night’s sleep

$\varepsilon_{ij}$: error component

As you can see, Model 2 tests whether someone’s affect after a night of sleep can be predicted by the last point of the previous day.

## Model 3

In a third model, we combined Model 1 and Model 2 to be able to compare the prediction of affect overnight to the prediction of affect during the day. This model was estimated using the following equation:

$Y_{ij}=\beta_{1i}*E_{ij}+\beta_{2i}*M_{ij}+\beta_{3i}*E_{ij}*X_{ij-1}+\beta_{4i}*M_{ij}*X_{ij-1}+\varepsilon_{ij}$

*Eq 3*

$Y_{ij}$*:* predicted affect for person i at time j

$X_{ij-1}$: affect of person i at time j-1

$E_{ij}$: dummy evening; all beeps that occurred in the evening, not after a night’s sleep

$M_{ij}$: dummy morning; all beeps which occurred in the morning, after a night’s sleep

$\beta_{1i}$: slope of dummy evening, or intercept for all beeps which occurred in the evening, not after a night’s sleep

$\beta_{2i}$: slope of dummy morning, or intercept for all beeps which occurred in the morning, after a night’s sleep

$\beta_{3i}$: slope of the lagged affect of a person at a certain beep for beeps that occurred in the evening, not after a night’s sleep

$\beta_{4i}$: slope of the lagged affect of a person at a certain beep for beeps that occurred in the morning, after a night’s sleep

$\varepsilon_{ij}$: error component

As you can see, Model 3 tests whether someone’s affect at one point of the day can predict their affect at the next point of the day and whether someone’s affect after a night of sleep can be predicted by the last point of the previous day.

## Model 4

In a fourth model, we tested whether having depressive symptoms is related to what extent affect is self-predictive during the day. This model was estimated using the following equation:

$$Y_{ij}=\beta_{1i}*E_{ij}+\beta_{2i}*E_{ij}*X_{ij-1}+\beta_{3i}*E_{ij}*CE{SD}_{i}+\beta_{4i}*E_{ij}*X_{ij-1}*CE{SD}_{i}+\varepsilon_{ij}$$

*Eq 4*

$Y_{ij}$: predicted affect for person i at time j

$X_{ij-1}$: affect of person i at time j-1

$CE{SD}_{i}$: score on CES-D questionnaire for person i

$E_{ij}$: dummy evening; all beeps that not occurred after a night’s sleep

$\beta_{1i}$: slope of dummy evening, or intercept for all beeps that not occurred after a night’s sleep

$\beta_{2i}$ : slope of the lagged affect of person i at a certain beep for beeps that not occurred after a night’s sleep

$\beta_{3i}$: slope of the CES-D score of person i for beeps that not occurred after a night’s sleep

$\beta_{4i}$: slope of the interaction between the lagged affect of person i at a certain beep and the CES-D score of person i, for beeps that not occurred after a night’s sleep

$\varepsilon_{ij}$: error component

As you can see, Model 4 tests whether level of depressive symptoms moderates the extent to which someone’s affect at one point of the day can predict their affect at the next point of the day.

## Model 5

In a fifth model, we tested whether having depressive symptoms is related to what extent affect is self-predictive overnight. This model was estimated using the following equation:

$$Y_{ij}=\beta_{1i}*M_{ij}+\beta_{2i}*M_{ij}*X_{ij-1}+\beta_{3i}*M_{ij}*CE{SD}_{i}+\beta_{4i}*M_{ij}*X_{ij-1}*CE{SD}_{i}+\varepsilon_{ij}$$

*Eq 5*

$Y_{ij}$: predicted affect for person i at time j

$X_{ij-1}$: lagged affect of person i at time j-1

$CE{SD}_{i}$: score on CES-D questionnaire for person i

$M_{ij}$: dummy morning; all beeps which occurred in the morning, after a night’s sleep

$\beta_{1i}$: slope of dummy morning, or intercept for all beeps which occurred after a night’s sleep

$\beta_{2i}$: slope of the lagged affect of person i at a certain beep for beeps that occurred after a night’s sleep

$\beta_{3i}$: slope of the CES-D score of person i for beeps that occurred after a night’s sleep

$\beta_{4i}$: slope of the interaction between the lagged affect of person i at a certain beep and the CES-D score of person i, for beeps that occurred after a night’s sleep

$\varepsilon_{ij}$: error component

As you can see, Model 5 tests whether level of depressive symptoms moderates the extent to which someone’s affect after a night of sleep can be predicted by the last point of the previous day.

## Model 6

In a sixth model, we tested whether subjective sleep quality relates to to what extent affect is self-predictive during the day. This model was estimated using the following equation:

$$Y_{ij}=\beta_{1i}*E_{ij}+\beta_{2i}*E_{ij}*X_{ij-1}+\beta_{3i}*E_{ij}*SQ_{ij}+\beta_{4i}*E_{ij}*X_{ij-1}*SQ_{ij}+\varepsilon_{ij}$$

*Eq 6*

$Y_{ij}$: predicted affect for person i at time j

$X_{ij-1}$: affect of person i at time j-1

$SQ_{ij}$: subjective sleep quality for person i at time j

$E_{ij}$: dummy evening; all beeps that not occurred after a night’s sleep

$\beta_{1i}$: slope of dummy evening, or intercept for all beeps that not occurred after a night’s sleep

$\beta_{2i}$ : slope of the lagged affect of person i at a certain beep for beeps that not occurred after a night’s sleep

$\beta_{3i}$: slope of the subjective sleep quality of person i at time j for beeps that not occurred after a night’s sleep

$\beta_{4i}$: slope of the interaction between the lagged affect of person i at a certain beep and the subjective sleep quality of person i at time j, for beeps that not occurred after a night’s sleep

$\varepsilon_{ij}$: error component

As you can see, Model 6 tests whether subjective sleep quality moderates the extent to which someone’s affect at one point of the day can predict their affect at the next point of the day.

## Model 7

In a seventh model, we tested whether subjective sleep quality relates to to what extent affect is self-predictive overnight. This model was estimated using the following equation:

$$Y_{ij}=\beta_{1i}*M_{ij}+\beta_{2i}*M_{ij}*X_{ij-1}+\beta_{3i}*M_{ij}*SQ_{ij}+\beta_{4i}*M_{ij}*X_{ij-1}*SQ_{ij}+\varepsilon_{ij}$$

*Eq 7*

$Y_{ij}$: predicted affect for person i at time j

$X_{ij-1}$: lagged affect of person i at time j-1

$SQ_{ij}$: subjective sleep quality for person i at time j

$M_{ij}$: dummy morning; all beeps which occurred in the morning, after a night’s sleep

$\beta_{1i}$: slope of dummy morning, or intercept for all beeps that occurred after a night’s sleep

$\beta_{2i}$ : slope of the lagged affect of person i at a certain beep for beeps that occurred after a night’s sleep

$\beta_{3i}$: slope of the subjective sleep quality of person i at time j for beeps that occurred after a night’s sleep

$\beta_{4i}$: slope of the interaction between the lagged affect of person i at a certain beep and the subjective sleep quality of person i at time j, for beeps that occurred after a night’s sleep

$\varepsilon_{ij}$: error component

As you can see, Model 7 tests whether subjective sleep quality moderates the extent to which someone’s affect after a night’s sleep can be predicted by the last point of the previous day.
